# Racial/ethnic disparities in the dose-response relationship between syndemic risk factors and increased gun carrying odds among male high school students in the United States

**DOI:** 10.1016/j.pmedr.2025.103267

**Published:** 2025-10-09

**Authors:** Marie-Claude Couture, Erin Grinshteyn, David Hemenway

**Affiliations:** a Department of Health Professions, School of Nursing and Health Professions, University of San Francisco, San Francisco, CA, USA; b Harvard Injury Control Research Center, Harvard T. H. Chan School of Public Health, Harvard University, Boston, MA, USA

**Keywords:** Gun carrying, High school students, Substance use, Violence, Mental health, Syndemic theory

## Abstract

**Objective:**

Substance use, mental health, and violence are co-occurring risks associated with gun carrying among adolescents. Using a syndemic framework, we examined the co-occurrence and cumulative effect of these risk factors on gun carrying in male adolescents and potential racial/ethnic disparities.

**Methods:**

We analyzed 2019–2023 Youth Risk Behavior Survey data with weighted multiple logistic regression models, controlling for age and sexual orientation. A syndemic risk factor variable was created by summing exposures to substance use, mental health, and victimization/violence. Interaction terms were tested, and models were stratified by race/ethnicity. A penalized maximum likelihood estimation (PMLE) regression was also applied to address potential instability due to the rarity of the outcome.

**Results:**

In multiple regression models, a significant dose-response relationship was found between the number of syndemic risk factors and the odds of gun carrying. Compared with male adolescents reporting 0–1 syndemic risk factors, the odds of gun carrying was more than five times higher among those with 2–3 syndemic risk factors (Adjusted Odds Ratio (AOR): 5.1, 95 % CI: 4.0, 6.6) and almost 17 times higher among those with 4+ syndemic risk factors (AOR: 17.3, 95 % CI: 12.9, 23.2). Stratified analyses revealed stronger effects among Black and Hispanic adolescents. Results were similar with PMLE regression.

**Conclusions:**

The odds of gun carrying increased as the number of syndemic risk factors increased, with notable racial/ethnic disparities. Understanding the syndemic risk factors associated with gun carrying and the disparities that exist is crucial for developing targeted prevention interventions.

## Highlights

• Substance use, mental health, and violence co-occur among students who carry guns.
• Gun carrying odds increased as the number of syndemic risk factors increased.
• Stronger effects were observed among Black and Hispanic male adolescents.
• Interventions must address the co-occurring syndemic risk factors of gun carrying.

## 1 Introduction

Adolescent firearm violence is a critical public health issue in the United States (US). Suicide and homicide are the second and fourth leading causes of mortality among adolescents aged 10–14 years, with 39 % of suicides and 85 % of homicides due to firearms (*Center for Disease Control and Prevention. WISQARS: Leading Causes of Death Visualization Tool*, 2021). Suicide and homicide are the third and second leading causes of death among those ages 15–19 years, with more than half (51 %) of suicides and the majority (95 %) of homicides due to firearms (*Center for Disease Control and Prevention. WISQARS: Leading Causes of Death Visualization Tool*, 2021). Compared with other high-income countries, the US has much higher adolescent firearm homicide and suicide rates – 31.1 and 10.6 times higher, respectively (Grinshteyn and Hemenway, 2019). Racial disparities persist, with firearm-related homicide rates highest among Non-Hispanic Black male adolescents, followed by Hispanic males, and Non-Hispanic White male adolescents (*Center for Disease Control and Prevention. Fatal Injury Reports, National, Regional and State*, 1981–2020, 2020). Yet, despite the prevalence of firearm mortality, relatively little is known about gun carrying in this population, one of the most important risk factors of firearm violence. Federal law generally prohibits minors under 18 from purchasing long guns (e.g., rifles, shotguns) and anyone under 21 from purchasing handguns. While exceptions allow minors to possess firearms under adult supervision for activities like hunting or target practice, in most cases, it remains illegal for anyone under 18 to own or possess a firearm.

Among US high school students, 1.9 % of females and 6.8 % of males had carried a gun on at least one day during the previous month (Simon et al., 2022). Perpetrators of firearm violence were more likely to use firearms against younger, Black males (Felson and Painter-Davis, 2012). Most studies examining racial/ethnic disparities in firearm carrying have found differences, however, the research is not consistent with respect to which racial/ethnic groups had a higher prevalence of firearm carrying (Simon et al., 2022; Kemal et al., 2018; Muula et al., 2008a; Vaughn et al., 2016; Jewett et al., 2021). Firearm carrying among adolescents can lead to several negative consequences. Research has found that firearm carrying was associated with suicide attempts overall and specifically among male adolescents (Simon et al., 2022; Romero et al., 2017; Woods et al., 1997). Carrying a gun has been associated with a higher risk for violence among adolescents (DuRant et al., 1995; Loughran et al., 2016), including physical fighting (Simon et al., 2022; Lowry, 1998) and firearm victimization (Watts, 2019).

Previous research has identified several risk factors for firearm carrying among adolescents, including violent behaviors, experience of victimization, and substance use, but few studies have examined racial/ethnic differences (Jewett et al., 2021; Docherty et al., 2019; Beardslee et al., 2018). Violent victimization (Simon et al., 2022; Jewett et al., 2021; Reid et al., 2017; DuRant, 1997), violent behavior (Simon et al., 2022), witnessing violence (Reid et al., 2017; Comer and Connolly, 2023; Harper et al., 2023), fighting (Simon et al., 2022; DuRant, 1997), and knowing victims of violence (Hemenway et al., 1996) have all been associated with adolescent firearm carrying. Black and Latino adolescents who had been threatened with a weapon were more likely to carry firearms at school (Semprevivo et al., 2020). Among male adolescents, threats or injury with a weapon and physical fights were both correlates of gun carrying (Simon et al., 2022; Singer et al., 2017). Substance use and mental health issues have been associated with firearm carrying in adolescents. Alcohol use and binge drinking (Simon et al., 2022; DuRant, 1997; Buschmann et al., 2017; Carter et al., 2013; Dukarm, 1996), marijuana use (Simon et al., 2022; Dukarm, 1996; Khubchandani and Price, 2018b), and smoking cigarettes (DuRant, 1997; Khubchandani and Price, 2018b) have been associated with firearm carrying among adolescents and emerging adults, as has illicit drug use (Simon et al., 2022; Carter et al., 2013; Dukarm, 1996; Khubchandani and Price, 2018b). Prescription medication use, specifically prescription opioid use, was associated with carrying a firearm (Buschmann et al., 2017; Carter et al., 2013). Among Hispanic and Black students, alcohol, drug, and tobacco use were strong correlates of weapon and gun carrying (Khubchandani and Price, 2018a; Khubchandani and Price, 2018b). Anxiety (Comer and Connolly, 2020), depression (Khubchandani and Price, 2018a; Khubchandani and Price, 2018b; Muula et al., 2008b), suicidal ideation (Simon et al., 2022; Khubchandani and Price, 2018a; Khubchandani and Price, 2018b; Muula et al., 2008b), and suicide attempts (Simon et al., 2022; Khubchandani and Price, 2018a; Khubchandani and Price, 2018b) were associated with weapon and gun carrying in adolescents. It is unclear whether the associations between substance use and mental health issues with firearm carrying differ within race/ethnicity groups.

Victimization, violence, substance use, and mental health issues often co-occur among adolescents, potentially reinforcing on another, and increasing the risk of firearm carrying in a phenomenon referred to as a syndemic. According to the *Syndemic Theory*, a syndemic involves a clustering of conditions (or risk factors) that interact together and mutually reinforce the risk of an adverse health outcome (Singer et al., 2017). Originally developed during the HIV/AIDS epidemic to examine the synergistic interaction of substance use, violence, and HIV infection, the syndemic framework has since been applied to study the syndemic of various health, psychological, and social issues in different contexts and populations (Mendenhall et al., 2022), including experiences of violence (Semple et al., 2017; Yang, 2023; Couture et al., 2020). Recently, experts have called for integrating the *Syndemic Theory* into firearm violence research to better understand how the clustering of afflictions in certain populations makes them more vulnerable to firearm violence (Lemke et al., 2022).

To our knowledge, no studies have used the *Syndemic Theory (*Singer et al., *2017**)* to examine the clustering of risk factors associated with adolescent gun carrying. Moreover, few studies have examined potential racial/ethnic differences in the multiple correlates of firearm carrying in adolescents. A recent scoping review called for more research examining firearm carrying across racial/ethnic subgroups among adolescents (Oliphant et al., 2019). This study aims to assess the co-occurrence and cumulative effect of syndemic risk factors associated with gun carrying among a nationally representative sample of male high school students by race/ethnicity. Better understanding the clustering syndemic of risk factors of gun carrying in adolescents and potential racial/ethnic disparities is imperative to tailor effective interventions to reduce firearm carrying among adolescents.

## 2 Methods

### 2.1 Data source and study participants

Data come from the 2019, 2021 and 2023 Youth Risk Behavior Survey (YRBS), a cross-sectional, nationally representative survey of U.S. high school students conducted biennially by the Centers for Disease Control and Prevention (CDC) (*Center for Disease Control and Prevention. YRBSS | Youth Risk Behavior Surveillance System | Data | Adolescent and School Health | CDC. May* 14, 2021). The sample included students in grades 9–12 from public and private schools, selected using a three-stage cluster sample design, with weights applied to account for nonresponse and ensure representativeness. Gun carrying is a rare outcome, thus, YRBS 2019, 2021 and 2023 datasets were combined to increase the sample size (*n* = 51,012) and statistical power. The YRBS was approved by the CDC's Institutional Review Board, and the de-identified data are publicly available.

### 2.2 Measures

Gun carrying was measured using the question: “During the past 12 months, on how many days did you carry a gun? (Do not count the days when you carried a gun only for hunting or for a sport, such as target shooting.)” and categorized as 1 “Yes, carried a gun one or more days” and 0 “No.” *Risk factors* included nine measures assessing substance use, mental health, and previous victimization and violence, which were selected based on the scientific literature and bivariate analysis. Substance use variables included binge alcohol drinking (yes/no), marijuana (yes/no), tobacco use in the last 30 days (yes/no), lifetime misuse of prescription pain medication (yes/no), and lifetime illicit drug use (heroin, cocaine, methamphetamines, ecstasy, hallucinogenic drugs, inhalants) (yes/no). Participants were asked if they had ever seriously considered suicide in the past 12 months (yes/no). They were also asked if they were in a physical fight (yes/no) and if someone threatened or injured them with a weapon (i.e., gun, knife, or club) on school property in the past 12 months. Experience of dating violence during the past year was assessed by combining physical dating violence and sexual dating violence (yes/no). A *syndemic risk factor variable* measuring the cumulative risk of exposure for each participant was created by summing the responses of these nine risk factors, creating a continuous score (range 0–9). The new syndemic risk score had a Cronbach's alpha of 0.7 and an average inter-item correlation of 0.19, indicating moderate reliability. A categorical variable was also created due to small sample sizes in some categories: 0–1 risk factor, 2–3 risk factors, 4+ risk factors. Syndemic risk factor subscale variables were also constructed representing the three separate dimensions: substance use, victimization, and mental health. The substance use subscale variable (range 0–5) summed five binary indicators: binge drinking, marijuana use, tobacco use, pain medication misuse, and illicit drug use. The victimization subscale (range 0–3) included experiences of being threatened or injured with a weapon, physical fight, and dating violence. The mental health subscale (range 0–1) included the single binary variable “considered attempting suicide”.

Sociodemographics included age and sexual orientation.

### 2.3 Statistical analyses

Analyses were restricted to male high school students (*n* = 25,518), excluding those with missing gun carrying data (*n* = 7741; 30.3 %). Frequencies and chi-square tests with complex survey weights were used to examine bivariate associations between all the syndemic risk factor variables and gun carrying. Multiple logistic regression models with complex survey weights, controlling for age and sexual orientation, were conducted to assess the additive association between the syndemic risk factor score (both continuous and categorical variables) and gun carrying. Effect modification by race/ethnicity was assessed, and models were stratified by three groups: White, Black, and Hispanic students. Due to the low prevalence of gun carrying, especially in stratified analyses, the statistical power was limited, and estimates were less stable. To address this, a sensitivity analysis was conducted using penalized maximum likelihood estimation (PMLE) regression models, a method suited for rare outcomes (King and Zeng, 2001), but which does not currently support complex survey weights. Additional multiple logistic regression models with complex survey weights examined associations between the three syndemic risk factor subscales (substance use, victimization, mental health) and gun carrying. Statistical analyses were performed using STATA 16.0 (STATA, College Station, TX).

## 3 Results

Of the male high school students, more than half (54.9 %) were White, 17.9 % were Black, and 27.3 % were Hispanic. Table 1 describes the sociodemographic characteristics, substance use behaviors, mental health, and victimization risk factors among White, Black, and Hispanic students. Over two-thirds of White male high school students (72.8 %) reported 0–1 syndemic risk factors, 19.4 % had 2–3 risk factors, and 7.8 % had 4+ syndemic risk factors. Among Black students, 77.3 % had 0–1 syndemic risk factors, 18.1 % had 2–3 risk factors, and 4.7 % had 4+ syndemic risk factors. Among Hispanic students, 76.5 % had 0–1 syndemic risk factors, 17.3 % had 2–3 risk factors, and 6.2 % 4+ syndemic risk factors.

**Table 1:** Prevalence of gun carrying by sociodemographic, substance use, mental health, and victimization risk factors among White, Black, and Hispanic male students in the United States (Youth Risk Behavior Survey 2019–2023).

Characteristics	**White Males (*n*** **=** **8.059)**			**Black Males (*n*** **=** **2623)**			**Hispanic Males (*n*** **=** **4002)**		
	Prevalence of characteristics	Prevalence of gun carrying	*p*-value	Prevalence of characteristics	Prevalence of gun carrying	p-value	Prevalence of characteristics	Prevalence of gun carrying	p-value
	%	%		%	%		%	%	
Total		5.0			7.7			5.8	
**Sociodemographics** Age									
*14 or less*	13.1	4.6		13.2	6.0		16.9	4.8	
*15*	24.8	4.5	<0.05	26.6	6.6	0.57	25.3	4.7	0.18
*16*	25.8	4.7		25.3	8.7		25.5	6.1	
*17*	23.6	4.2		23.9	8.8		22.8	5.6	
*18 or more*	12.7	8.0		11.0	8.2		9.5	9.1	

Sexual Orientation									
*Heterosexual*	89.3	4.7	0.54	90.6	6.7	<0.05	90.7	5.4	0.44
*LGB/Other*	10.7	4.1		9.4	12.3		9.3	6.8	

**Substance use** Recent binge alcohol drinking									
*No*	87.1	3.1	<0.01	96.3	5.1	<0.01	92.7	3.4	<0.01
*Yes*	12.9	12.7		3.7	39.5		7.4	23.0	
Recent tobacco use									
*No*	94.0	3.9	<0.01	97.6	6.0	<0.01	96.3	4.4	<0.01
*Yes*	6.0	16.5		2.5	61.4		3.7	34.5	
Recent marijuana use									
*No*	82.6	3.6	<0.01	82.4	3.6	<0.01	83.8	3.0	<0.01
*Yes*	17.4	10.9		17.7	24.3		16.2	17.3	
Lifetime prescription pain medication misuse									
*No*	90.6	3.6	<0.01	89.0	5.7	<0.01	88.8	3.9	<0.01
*Yes*	9.4	17.4		11.0	22.3		11.3	19.6	
Lifetime illicit drug use									
*No*	87.5	3.7	<0.01	93.0	5.8	<0.01	89.6	3.9	<0.01
*Yes*	12.5	13.5		7.1	33.7		10.4	21.7	

**Mental Health** Considered attempting suicide									
*No*	85.3	4.4	<0.01	88.9	6.3	<0.01	87.5	4.9	<0.01
*Yes*	14.7	8.0		11.1	16.9		12.5	11.6	

**Victimization/violence** Threatened or injured by a weapon									
*No*	93.2	4.0	<0.01	90.2	5.2	<0.01	91.5	4.2	<0.01
*Yes*	6.8	17.3		9.8	31.3		8.5	22.7	
In a physical fight									
*No*	75.6	2.3	<0.01	69.6	3.4	<0.01	76.5	3.0	<0.01
*Yes*	24.4	12.9		30.4	15.8		23.5	13.6	
Experienced dating violence									
*No*	94.2	3.9	<0.01	92.5	5.1	<0.01	94.3	4.2	<0.01
*Yes*	5.8	19.4		7.5	36.4		5.7	30.0	

**Syndemic risk factors** Number of syndemic risk factors (continuous)									
*0*	50.5	1.7	<0.01	49.9	1.2	<0.01	52.7	1.1	<0.01
*1*	22.3	4.8		27.3	5.9		23.8	4.2	
*2*	12.7	5.8		12.7	14.4		11.5	8.9	
*3*	6.7	9.3		5.4	26.9		5.8	18.2	
*4*	3.5	12.7		1.8	26.6		3.1	25.8	
*5*	2.2	17.1		1.2	49.1		1.3	28.5	
*6*	1.1	33.3		0.5	72.8		0.8	40.3	
*7*	0.8	47.7		0.7	56.0		0.5	47.2	
*8*	0.1	53.8		0.4	96.8		0.2	76.1	
*9*	0.1	63.3		0.1	81.2		0.3	74.0	
Number of syndemic risk factors (categorical)									
*0–1*	72.8	2.6	<0.01	77.3	2.8	<0.01	76.5	2.1	<0.01
*2–3*	19.4	7.0		18.1	18.2		17.3	12.0	
*4+*	7.8	21.7		4.7	48.4		6.2	33.5	

Note Chi-square tests with complex survey weights; Syndemic risk score: sum variable of the nine syndemic risk factors (substance use, mental health, victimization/violence); LGB: Lesbian. Gay, Bisexual

Almost 7 % (6.8 %; *n* = 1205) of male students reported gun carrying in the past year. Gun carrying prevalence was 5.0 % among White, 7.7 % among Black, and 5.8 % among Hispanic males (*p* < 0.01) (Table 1). Gun carrying was higher among all students who reported recent binge alcohol drinking, smoking, marijuana use, lifetime misuse of prescription pain medication, and illicit drug use than (*p* < 0.01). White, Black, and Hispanic adolescents who had considered attempting suicide reported more gun carrying than those who did not. Gun carrying was also higher among all students who had been threatened or injured with a weapon, were in a physical fight, and had experienced dating violence (p < 0.01). Gun carrying increased as the number of syndemic risk factors increased for all male students, with stronger effects among Black and Hispanic students (Table 1). Among adolescents reporting 4+ syndemic risk factors, the prevalence of gun carrying was 21.7 % among White students, 48.4 % among Black, and 33.5 % among Hispanic students.

In the bivariate associations, all nine syndemic risk factors were highly correlated with gun carrying in male adolescents (Table 2). Associations were observed between all substance use, mental health, and violence victimization variables, supporting the clustering of risk factors.

**Table 2:** Bivariate associations between substance use, mental health, and victimization syndemic risk factors and gun carrying in among male high school students in the United States (Youth Risk Behavior Survey 2019–2023).

	Current binge alcohol drinking	Current tobacco use	Current marijuana use	Lifetime prescription pain medication misuse	Lifetime illicit drug	Considered attempting suicide	Threatened or injured by a weapon	In a physical fight	Experienced dating violence
	OR (95 % CI)	OR (95 % CI)	OR (95 % CI)	OR (95 % CI)	OR (95 % CI)	OR (95 % CI)	OR (95 % CI)	OR (95 % CI)	OR (95 % CI)
Gun carrying last year	5.8 (4.4,7.8)	7.2 (5.2,9.9)	5.3 (4.2,6.6)	5.8 (4.6,7.3)	5.5 (4.4,6.7)	2.3 (1.9,2.8)	6.6 (5.3,8.2)	6.1 (4.9,7.6)	7.6 (5.9,10.0)
Recent binge alcohol drinking	–	23.2 (18.3,29.3)	11.3 (9.4,13.5)	3.9 (3.1,4.8)	8.1 (6.8,9.7)	2.3 (1.9,2.8)	3.1 (2.5,3.9)	3.6 (3.0,4.2)	4.48 (3.6,6.4)
Recent tobacco use	–	–	12.0 (9.3,15.4)	7.4 (5.7,9.5)	13.7 (11.0,17.1)	3.7 (2.9,4.6)	3.9 (3.0,5.2)	4.6 (3.6,5.9)	6.1 (4.5,8.2)
Recent marijuana use	–	–	–	3.8 (3.2,4.4)	9.4 (7.9,11.2)	2.8 (2.4,3.2)	2.7 (2.3,3.2)	3.3 (2.8,3.8)	3.9 (3.2,4.8)
Lifetime prescription pain medication misuse	–	–	–	–	7.9 (6.7,9.4)	3.6 (3.2,4.2)	4.5 (3.7,5.4)	2.7 (2.2,3.2)	4.3 (3.5,5.3)
Lifetime illicit drug	–	–	–	–	–	4.0 (3.4,4.6)	4.4 (3.7,5.2)	3.5 (3.0,4.1)	5.2 (4.2,6.5)
Considered attempting suicide	–	–	–	–	–	–	3.5 (3.0,4.0)	2.3 (2.1,2.7)	4.7 (4.0,5.6)
Threatened or injured by a weapon	–	–	–	–	–	–	–	5.0 (4.2,5.7)	7.5 (6.0,9.4)
In a physical fight	–	–	–	–	–	–	–	–	4.0 (3.3,4.8)
Experienced dating violence	–	–	–	–	–	–	–	–	–

A significant dose-response relationship was observed where the odds of firearm carrying increased as the syndemic risk factor score increased (Table 3). Stronger associations were observed in Black (Adjusted Odds Ratio (AOR) = 2.3; 95 % CI: 1.9, 2.7) and Hispanic students (AOR = 2.0; 95 % CI: 1.8, 2.2) compared with White students (AOR = 1.7; 95 % CI: 1.5, 1.9). Among White students, the odds of gun carrying were higher among those who had 2–3 syndemic risk factors (AOR = 2.9; 95 % CI: 1.9, 4.4) and those who had 4+ risk factors (AOR = 11.0; 95 % CI: 6.8, 17.9). The strongest addictive associations were observed among Black and Hispanic adolescents. The odds of gun carrying were nine times higher among Black students who reported 2–3 syndemic risk factors (AOR = 9.1; 95 % CI: 5.1, 16.3) and 42 times higher among those who reported 4+ risk factors (AOR = 41.9; 95 % CI: 22.9, 76.7). Among Hispanic students, the odds of gun carrying were more than seven times higher among those who had 2–3 syndemic risk factors (AOR = 7.4; 95 % CI: 4.9, 11.0) and more than 26 times higher among those who had 4+ risk factors (AOR = 26.1; 95 % CI: 17.3, 39.4). Using PMLE multiple regression models for rare outcomes, similar trends and differences across race/ethnicity were observed, but with more precise estimates (Table 4).

**Table 3:** Weighted multiple regression models for the associations between syndemic risk factors and gun carrying among male high school students in the United States (Youth Risk Behavior Survey 2019–2023).

	**All male students** **(*n*** **=** **17,777)**	**White students** **(n** **=** **8.059)**	**Black students** **(n** **=** **2623)**	**Hispanic students** **(n** **=** **4002)**
	AOR (95 % CI)	AOR (95 % CI)	AOR (95 % CI)	AOR (95 % CI)
**Syndemic risk factors**				
Continuous variable	1.8 (1.7, 1.9)	1.7 (1.5, 1.9)	2.3 (1.9, 2.7)	2.0 (1.8, 2.2)
Categorical variable				
*0–1*	1.0	1.0	1.0	1.0
*2–3*	5.1 (4.0, 6.6)	2.9 (1.9, 4.4)	9.1 (5.1, 16.3)	7.4 (4.9, 11.0)
*4+*	17.3(12.9, 23.2)	11.0 (6.8, 17.9)	41.9 (22.9, 76.7)	26.1 (17.3, 39.4)


	**All male students** **(n** **=** **17,777)**	**White students** **(n** **=** **8.059)**	**Black students** **(n** **=** **2623)**	**Hispanic students** **(n** **=** **4002)**
	AOR (95 % CI)	AOR (95 % CI)	AOR (95 % CI)	AOR (95 % CI)
**Syndemic risk factors**				
Continuous variable	1.8 (1.7, 1.8)	1.7 (1.6–1.7)	2.1 (1.9–2.3)	1.9 (1.8–2.1)
Categorical variable				
*0–1*	1.0	1.0	1.0	1.0
*2–3*	5.1 (4.4, 6.0)	3.3 (2.6, 4.2)	7.6 (5.4, 10.6)	6.3 (4.5, 8.8)
*4+*	14.9 (12.6, 17.6)	10.2 (8.0, 13.1)	27.4 (17.6, 42.6)	23.3 (16.4, 33.2)

Note. AOR: Adjusted odds ratio; CI: Confidence Interval; Unweighted penalized maximum likelihood estimation regression models adjusted for age and sexual orientation; Syndemic risk score: sum variable of the nine syndemic risk factors (substance use, mental health, victimization/violence)

Among White, Black, and Hispanic students, higher scores on the substance use, victimization, and mental health subscales were each significantly associated with increased odds of gun carrying (Supplemental Table). Victimization showed the strongest associations overall (AOR = 3.7; 95 % CI: 3.3, 4.2), particularly among Black (AOR = 4.3; 95 % CI: 3.3, 5.5) and Hispanic (AOR = 3.7; 95 % CI: 2.9, 4.7) students. Substance use and mental health were also significant across all racial/ethnic groups, with the highest adjusted odds ratios for gun carrying seen among Black students.

## 4 Discussion

Firearm carrying among adolescents is a critical public health issue that has been understudied, despite its potential for significant negative consequences and its illegality for those under 18 outside of limited exceptions (e.g., target practice or supervised hunting). Yet, over 85 % of the entire sample and 85 % of those who carried a firearm in this sample were under 18 years of age. Moreover, the YRBS dataset explicitly excludes legal exceptions (e.g., supervised use for hunting or target practice) in its firearm-carrying question, underscoring the relevance of these findings.

This is the first study to use a syndemic approach to examine the cumulative effect of co-occurring risk factors on gun carrying among male adolescents in the US. We identified a syndemic of risk factors – substance use, violence, and mental health - clustering together among male adolescents and increasing the odds of gun carrying. While half of the adolescents reported no syndemic risk factors, those who did (49 %) accounted for a substantial majority (86 %) of the gun carriers. Furthermore, our research uncovers a dose-response relationship, with gun carrying increasing as the number of syndemic risk factors increased. Our study also revealed racial/ethnic disparities, with stronger cumulative effects of the syndemic risk factors on gun carrying among Black and Hispanic male adolescents. These findings extend existing research by demonstrating the syndemic effect of these risk factors on adolescent gun carrying.

Our findings support previous research showing that substance use, mental health, and violence strongly correlate with firearm carrying in youth. Alcohol use and misuse have been associated with gun and weapon carrying among adolescents and young adults (DuRant, 1997; Buschmann et al., 2017; DuRant et al., 1999; Vaughn et al., 2019). Black and Hispanic adolescent males who reported recent alcohol use were more likely to carry a gun and other weapons (Khubchandani and Price, 2018a; Khubchandani and Price, 2018b). Marijuana use was also correlated with adolescent gun carrying overall (DuRant et al., 1999; Vaughn et al., 2019) and specifically among Black and Hispanic adolescent males (Khubchandani and Price, 2018a; Khubchandani and Price, 2018b). Prescription pain medication is another important correlate of adolescent firearm carrying (Buschmann et al., 2017) with those misusing prescription opioids being five times more likely to carry a gun (Bhatia et al., 2020). Comer et al (Comer and Connolly, 2020) found no statistically significant associations between alcohol, marijuana, and hard drug use and gun carrying among Black, Hispanic, and White adolescent males, possibly due to low statistical power. Previous studies have also linked depression, suicidal ideation, and suicide attempts to weapon and gun carrying among adolescents (Simon et al., 2022; Muula et al., 2008a; Khubchandani and Price, 2018a; Khubchandani and Price, 2018b).

Adolescents who experienced violence may carry guns for self-protection, a known motivation for firearm carrying (Hemenway et al., 1996; Carter et al., 2013). Previous experience of violence is an important risk factor for adolescent firearm carrying (Simon et al., 2022; Reid et al., 2017), with being threatened or injured with a weapon associated with seven times the odds of firearm carrying among Black adolescents and more than six times among Hispanic adolescents (Khubchandani and Price, 2018a; Khubchandani and Price, 2018b). Comer et al (Comer and Connolly, 2020) found that being threatened, attacked, and bullied were strong correlates of gun carrying among Hispanic adolescents, but not among Black and White youth. The effect of dating violence on firearm carrying has been understudied, though one study found that students who had been raped were 1.7 times more likely to carry a weapon (Muula et al., 2008a). Forced sex was also an important correlate of firearm carrying among Hispanic and Black adolescent males (Khubchandani and Price, 2018a; Khubchandani and Price, 2018b).

This study used a large national sample of adolescent males, but several limitations should be considered. The relatively low number of firearm carriers reduced the statistical power and precision of the estimates. Given the cross-sectional design, we cannot determine the directionality of the relationships or rule out the possibility of reverse causation. Self-reported data on firearm carrying, substance use, mental health, and victimization may be subject to recall and social desirability biases. However, the YRBS questionnaire has demonstrated strong validity and reliability, and any bias is likely to result in underreporting of stigmatized behaviors, potentially biasing our results toward the null. Residual confounding may still exist as some potentially important confounders were not measured, including family income, gang membership, and neighborhood disadvantage. The YRBS dataset does not include fully geocoded data, which precludes analysis of community-level contextual factors. There is a potential selection bias, as adolescents who were home-schooled, did not attend, or dropped out of school were not sampled in this study. Youth who did not attend or had dropped out of school may be more likely to engage in high-risk behaviors, including gun carrying.

Despite these limitations, our study highlights the need to better understand the clustering syndemic of risk factors of gun carrying in adolescents and the racial/ethnic differences. Future research using longitudinal study designs is needed to better understand the temporality of the associations between syndemic risk factors and firearm carrying. Studies using larger sample sizes of firearm carrying in diverse ethnic/racial adolescents would be necessary to improve statistical power and increase the generalizability of the findings. Studies should investigate the roles of community-level factors in adolescent gun carrying using geocoded datasets. Finally, mixed-method research studies could help better understand when, where, and why adolescents carry firearms.

Our findings have important implications for public health and may inform future interventions to reduce adolescent firearm carrying and the resulting consequences. While little is known about how to prevent adolescent firearm carrying (Price and Khubchandani, 2019), primary prevention aims to reduce adolescents' access to a firearm. Child access prevention (CAP) laws are associated with a reduction in gun carrying among adolescents (Anderson and Sabia, 2018). Even when firearms are locked, adolescents still report being able to access them easily (Salhi et al., 2021), thus, CAP laws are insufficient. Adolescents also acquire firearms from the black market, thefts, and friends (Webster et al., 2014). Stronger state and local gun laws, which have been associated with a reduction in adolescent gun carrying (Dong and Wilson, 2022; Xuan and Hemenway, 2015), could be employed to prevent access to illegal guns. Within schools, hardening facilities to make them less susceptible to violence has been employed, though these efforts are mostly aimed at preventing external threats and largely have been ineffective (Price and Khubchandani, 2019). Comprehensive school-based interventions addressing the co-occurrence of syndemic risk factors associated with adolescent gun carrying, such as substance use, mental health, and violence, could reduce these risk factors and the resulting firearm carrying. A variety of school-based approaches to reduce dating violence, tobacco, alcohol, and drug use have been shown to be effective (Das et al., 2016; Lundgren and Amin, 2015). Integrating evidence-based violence prevention interventions into school-based substance use programs for adolescents could effectively address the clustering of risk factors and firearm violence, helping to prevent multiple serious public health issues among youth.

## 5 Conclusion

Firearm carrying is a critical public health issue among adolescents in the US, contributing to injuries and being a leading cause of mortality (*Center for Disease Control and Prevention. WISQARS: Leading Causes of Death Visualization Tool*, 2021). Our findings reveal that risk factors, such as substance use, mental health, victimization, and violence, often co-occur among male adolescents and interact to increase firearm carrying, with notable disparities in these associations. A deeper understanding of these syndemic risk factors is essential to identify at-risk youth and develop effective, comprehensive prevention strategies to reduce firearm carrying.

## CRediT authorship contribution statement

**Marie-Claude Couture:** Writing – original draft, Visualization, Methodology, Formal analysis, Conceptualization. **Erin Grinshteyn:** Writing – review & editing, Conceptualization. **David Hemenway:** Writing – review & editing, Supervision.

## Funding sources

This research did not receive any specific grants from funding agencies in the public, commercial, or not-for-profit sectors.

## Declaration of competing interest

The authors declare that they have no known competing financial interests or personal relationships that could have appeared to influence the work reported in this paper.

## Data Availability

The data used in this study comes from a publicly available anonymized dataset: https://www.cdc.gov/yrbs/index.html

## Appendix A Supplementary data

Supplementary material: Adjusted Odds Ratios of Gun Carrying by Subscales. Image 1
