# Supplementary material for: Racial/ethnic disparities in the dose-response relationship between syndemic risk factors and increased gun carrying odds among male high school students in the United States
Source: Prev Med Rep. 2025 Oct 9;59:103267. doi: 10.1016/j.pmedr.2025.103267 (PMC12552995; doi:10.1016/j.pmedr.2025.103267)
Supplement: Supplementary file 1 — Supplementary material: Adjusted Odds Ratios of Gun Carrying by Subscales. [file mmc1.docx]

| **Supplemental table. Adjusted odds ratios of gun carrying by subscales of syndemic risk factors among male high school students in the United States (Youth Risk Behavior Survey 2019-2023)** | | | | |
| --- | --- | --- | --- | --- |
|  | **All male students**  **(n=17,777)** | **White students**  **(n=8,059)** | **Black students**  **(n=2,623)** | **Hispanic students**  **(n=4,002)** |
|  | AOR (95% CI) | AOR (95% CI) | AOR (95% CI) | AOR (95% CI) |
| **Syndemic risk factor subscales** |  |  |  |  |
| Substance use | 2.1 (1.9, 2.3) | 1.8 (1.6, 2.1) | 3.0 (2.3, 3.8) | 2.4 (2.1, 2.8) |
|  |  |  |  |  |
| Victimization/violence | 3.7 (3.3, 4.2) | 3.6 (2.9, 4.3) | 4.3 (3.3, 5.5) | 3.7 (2.9, 4.7) |
|  |  |  |  |  |
| Mental health | 2.4 (1.9, 3.0) | 2.2 (1.6, 3.0) | 3.3 (2.2, 5.0) | 2.6 (1.6, 4.3) |
|  |  |  |  |  |
| Note. AOR: Adjusted odds ratio; CI: Confidence Interval; Weighted multiple regression models adjusted for age and sexual orientation | | | | |
